# Pulmonary thromboembolism in conjunction with intracavitary thrombus caused by severe acute respiratory syndrome coronavirus-2 infection in a patient living with human immunodeficiency virus

**DOI:** 10.1590/0037-8682-0157-2021

**Published:** 2021-06-02

**Authors:** Ana Carolina Veronese Silva, Paulo André Bispo Machado-Junior, Leticia Bressan Anizelli, Rebecca Benicio Stocco, Lidia Ana Zytynski Moura, Jarbas da Silva Motta, Gustavo Gavazzoni Blume

**Affiliations:** 1 Pontifícia Universidade Católica do Paraná, Escola de Medicina, Curitiba, PR, Brasil.; 2 Hospital Marcelino Champagnat, Centro de Ensino, Pesquisa e Inovação, Curitiba, PR, Brasil.

**Keywords:** COVID-19, Blood coagulation disorders, HIV Infections

## Abstract

Approximately one-third of patients with coronavirus disease 2019 (COVID-19) present with coagulation disorders and hematological changes. However, the clinical manifestations of COVID-19 and prognoses of people living with human immunodeficiency virus (HIV) remain controversial. This study reports the case of a 27-year-old HIV-infected man who regularly used antiretroviral medications, had no other comorbidities and was admitted for acute respiratory distress syndrome caused by COVID-19. Complementary examinations during hospitalization revealed a diagnosis of pulmonary thromboembolism in association with an intracavitary thrombus.

## INTRODUCTION

Thromboembolic events caused by severe acute respiratory syndrome coronavirus-2 (SARS-CoV-2) have become widespread, especially in patients with elevated D-dimer levels, advanced age, history of cardiovascular disease, severe symptoms and patients in need of intensive care unit (ICU) support^1^. The pathogenesis of these events involves endothelial dysfunction and a hypercoagulable state unleashed by direct invasion of the virus in endothelial cells, microvascular inflammation and high expression of proinflammatory markers^2^.

In people living with human immunodeficiency virus (HIV), the clinical manifestations and prognosis of SARS-CoV-2 infection remain controversial. Herein, we present the case of a 27-year-old man with HIV infection who was admitted with acute respiratory distress syndrome in association with an intracavitary thrombus in the right atrium and a pulmonary embolism caused by coronavirus disease 2019 (COVID-19).

## CASE REPORT

**FIGURE 1:**
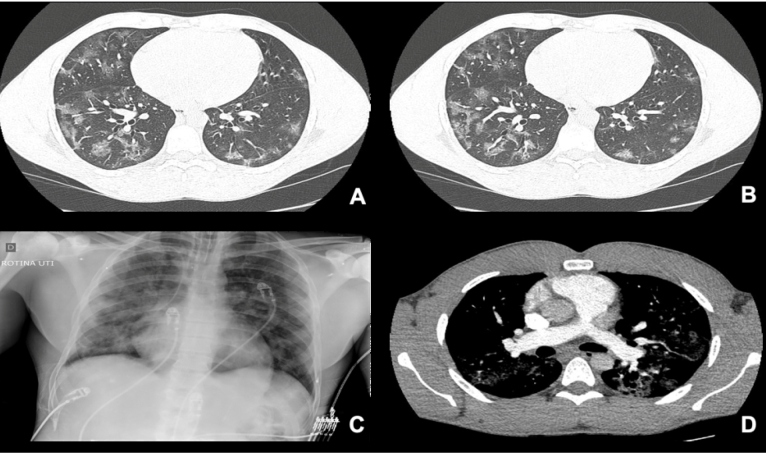
Images obtained at admission. CT scan demonstrating multiple lobe ground-glass opacities **(A and B)**, chest radiograph with peripheral opacities **(C)**, and CTPA with no signs of thromboembolism **(D)**. **CT:** computed tomography; **CTPA:** computed tomography pulmonary angiogram.

**FIGURE 2:**
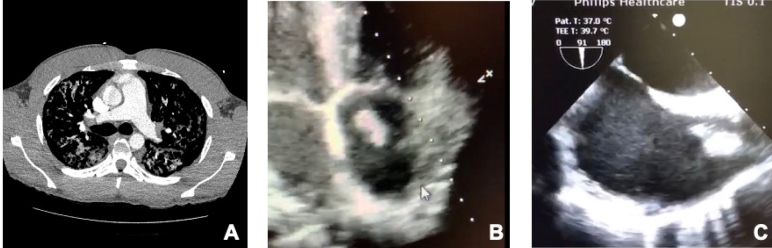
CTPA with filling defects in the pulmonary arteries bilaterally **(A)**, transthoracic **(B)**, and transesophageal **(C)** echocardiography showing thrombus in the right atrium. **CTPA:** computed tomography pulmonary angiogram.

A 27-year-old man with HIV infection for 12 years and regular use of antiretroviral medications (nevirapine 200 mg by mouth once daily, lamivudine 150 mg by mouth once daily, and zidovudine 300 mg by mouth once daily) and no other comorbidities was admitted to the emergency room on July 20, 2020 complaining of dyspnea and persistent hypoxia with a positive PCR test result for SARS-CoV-2 for 10 days, with constitutional symptoms (fever and myalgia) for about 12 days. His vital signs revealed a temperature of 37.3°C and indicated tachypnea (respiratory rate, 22 breaths per min), tachycardia (heart rate, 104 beats per min), hypertension (blood pressure, 130/77 mmHg), and hypoxia (oxygen saturation, 87%). On physical examination, the patient presented in good condition, with no obvious sounds heard during pulmonary examination. The laboratory test results were as follows: lactic acid level (1.9 mg/dL), D-dimer level (2.231 μg/L), C- reactive protein level (109.8 mg/dL), arterial blood gases (pH: 7.53; PaO_2_: 76 mmHg; PaCO_2_: 34 mmHg; HCO_3_: 28 mEq/L), neutrophil/lymphocyte ratio (18.2), creatinine (0.96 mg/dL), blood cell counts (3.8 10^6^/microliter) and hemoglobin (14.5 g/dL). His viral load was undetectable, and his CD4+ cell count was 324 cells/µL. Chest computed tomography (CT) images demonstrated ground-glass opacity, and the CT pulmonary angiogram (CTPA) revealed no signs of a pulmonary thromboembolism (Figure 1). The patient was prescribed enoxaparin (60 mg daily), ceftriaxone (2 g daily), and dexamethasone (6 mg daily). The patient was initially submitted to noninvasive ventilation, but developed progressive worsening of his breathing pattern requiring orotracheal intubation, sedation, and admission to the ICU. After nine days, the patient was discharged from the ICU; however on August 6, 2020, he was readmitted due to a new episode of dyspnea, tachycardia, and fever. A transthoracic echocardiogram revealed the presence of a right atrial thrombus, the new CTPA demonstrated filling defects in the left pulmonary artery compatible with a pulmonary thromboembolism (Figure 2). The patient received enoxaparin (80 mg twice daily), as well as linezolid (600 mg by mouth twice daily), empirical anidulafungin (100 mg by mouth daily), and sulfamethoxazole/trimethoprim (400 mg intravenous four times daily) due to possible pulmonary sepsis, which was suspected due to the increases in proinflammatory markers. He presented with hemodynamic instability, received noradrenaline (20 ml/h), and was again submitted to orotracheal intubation with pronation, which was unsuccessful. Extracorporeal membrane oxygenation was performed from August 11, 2020 until August 31, 2020. During this time, the patient also presented with moderate bleeding from the nasal and oral cavities, requiring a blood transfusion (11 U of packed red blood cells and 18 U of plasma) in the next few days. Additionally, he presented with a new episode of fever on August 21, 2020, and *Pseudomonas aeruginosa* and *Escherichia coli* were detected in pulmonary and tracheal aspirate cultures. Moreover, multisensitive *Candida parapsilosis* was detected in the blood culture and fluconazole (200 mg intravenous daily) was administered. The patient was discharged from the ICU to the infirmary on September 18, 2020 and on September 21, 2020 he was discharged from the hospital with prescriptions for fluconazole (200 mg), sulfamethoxazole/trimethoprim (400 mg), rivaroxaban (15 mg), omeprazole (40 mg), analgesics, and his regular antiretroviral medications. The patient continued in the outpatient follow-up clinic and underwent physiotherapy at home.

This research was approved by the Pontifícia Universidade Católica do Paraná's Research Ethics Committee, under the opinion number 4.356.426. Furthermore, the patient signed an informed consent agreeing with this research.

## DISCUSSION

In the context of the COVID-19 pandemic, comorbidities such as coronary heart disease, diabetes, and neoplasms are considered risk factors for developing the severe form of the disease^3^. Despite this, there is still a lack of data evaluating the role of SARS-CoV-2 infection in patients living with HIV; thus, the possible relationship between the risk of death and SARS-CoV-2 infection in these patients is still not elucidated^4^.

A recent cohort study conducted in South Africa demonstrated that the risk of mortality from COVID-19 in HIV-infected patients was almost twice that of noninfected patients^5^. On the other hand, observational studies in the United States and Germany showed no significant impact on length of hospitalization or clinical outcomes in patients coinfected with SARS-CoV-2 and HIV in comparison to patients without HIV^6^ ^,^ ^7^. In Brazil, there is only one case report of a patient with HIV coinfected with SARS-CoV-2, which had a good clinical outcome without the need for invasive ventilation or ICU support^8^.

In the present case, the patient received pharmacological prophylaxis against thromboembolic events and did not present with the suggestive manifestations of a pulmonary thromboembolism on the initial CT scan (Figure 1C). However, the patient developed acute respiratory distress syndrome with pulmonary sepsis and thrombotic manifestations observed through capillary refill failures on chest CT angiography and an intracardiac thrombus on the echocardiogram (Figure 2). 

Previous reports have already demonstrated the presence of intracardiac thrombi detected through echocardiography in the evaluation of patients with severe COVID-19^9^; this has led to a consensus about the importance of echocardiographic images for the evaluation and monitoring of these patients, since thrombotic events in patients with COVID-19 are related to a worse prognosis^10^.

These thrombotic events are mostly related to the presence of SARS-CoV-2 in the airways, which can unleash an innate immune response mediated initially by macrophages that generate a disproportionate immune response causing endotheliitis and the formation of microthrombi. Furthermore, the complement system is activated, causing the “cytokine storm” phenomenon, which aggravates the endothelial injury and unleashes the systemic inflammatory response syndrome^11^.

Regardless, in patients living with HIV, cellular immunity deficiency can paradoxically be a protective factor against the cytokine storm in patients with COVID-19^7^ ^,^ ^12^. Shalev et al., during the evaluation of the clinical outcomes of 31 HIV-infected patients coinfected with SARS-CoV-2, hypothesized that the absence of T-cell activation may mitigate the immunopathological effects observed in patients with COVID-19. Thus, SARS-CoV-2 infection would not be considered an opportunistic disease in patients with uncontrolled HIV or patients progressing to the AIDS phase^12^.

In the present case, we showed that despite the initial prophylaxis against thrombotic manifestations and cellular immunity deficiency, an HIV-infected patient coinfected with SARS-CoV-2 progressed to the severe form of the disease.
